# Correction to “Structure and Biosynthesis of Hectoramide B, a Linear Depsipeptide from Marine Cyanobacterium Moorena producens JHB Discovered via Coculture with *Candida albicans*”

**DOI:** 10.1021/acschembio.4c00739

**Published:** 2024-11-26

**Authors:** Thuan-Ethan Ngo, Andrew Ecker, Byeol Ryu, Aurora Guild, Ariana Remmel, Paul D. Boudreau, Kelsey L. Alexander, C. Benjamin Naman, Evgenia Glukhov, Nicole E. Avalon, Vikram V. Shende, Lamar Thomas, Samira Dahesh, Victor Nizet, Lena Gerwick, William H. Gerwick

The authors regret that several figures in the Supporting Information (sections S6, S7, S9, S12, S15) did not transfer properly and were represented by red X’s. The Supporting Information with this Correction replaces the previously published file.

## Supporting Information Available

NMR and other spectroscopic data and spectra for hectoramide B, additional analysis of the biosynthetic gene cluster for hectoramide B production, data from the LC–MS analysis of the preliminary coculture and monoculture experiments, and data generated from evaluation of hectoramide A for anti-*Candida* activity (PDF)

