# Supplementary material for: Structure and Biosynthesis of Hectoramide B, a Linear Depsipeptide from Marine Cyanobacterium Moorena producens JHB Discovered via Coculture with Candida albicans
Source: ACS Chem Biol. 2024 Feb 8;19(3):619–28. doi: 10.1021/acschembio.3c00391 (PMC10949194; doi:10.1021/acschembio.3c00391)
Supplement: Supplementary file 1 — cb3c00391_si_001.pdf [file cb3c00391_si_001.pdf]

SUPPLEMENTARY INFORMATION

Structure and Biosynthesis of Hectoramide B, a Linear Depsipeptide from the Marine Cyanobacterium *Moorena producens* JHB Discovered via Co-culture with *Candida albicans*

Thuan-Ethan Ngo[1], Andrew Ecker[1,2], Byeol Ryu,[1] Aurora Guild[1], Ariana Remmel[1], Paul D. Boudreau[1,3], Kelsey L. Alexander[1,4], C. Benjamin Naman[1,5], Evgenia Glukhov[1], Nicole E. Avalon[1], Vikram V. Shende[1], Lamar Thomas,[6] Samira Dahesh,[6] Victor Nizet,[6,7] Lena Gerwick[1], William H. Gerwick*[1,7]

[1]Center for Marine Biotechnology and Biomedicine, Scripps Institution of Oceanography, University of California San Diego, 9500 Gilman Drive, La Jolla, CA, 92093, USA

[2]Department of Pharmaceutical Chemistry, Cardiovascular Research Institute, University of California San Francisco, San Francisco, CA, 94143, USA

[3]Department of BioMolecular Sciences, University of Mississippi, School of Pharmacy, University, MS, 38677, USA

[4]Department of Chemistry, University of California San Diego, 9500 Gilman Drive, La Jolla, CA 92093, USA

[5]Department of Science and Conservation, San Diego Botanic Garden, 300 Quail Gardens Drive, Encinitas, CA, 92024, USA

[6]Department of Pediatrics, University of California, San Diego, 9500 Gilman Drive, La Jolla, CA 92093, USA

[7]Skaggs School of Pharmacy and Pharmaceutical Sciences, University of California San Diego, 9500 Gilman Drive, La Jolla, CA, 92093, USA

Corresponding Author: W. H. Gerwick, email: wgerwick@health.ucsd.edu

Table of Contents

**Spectroscopic Data for Hectoramide B (1)**		**Page**
**Table S1.**	**NMR data summary for hectoramide B (1).**	**S3**
**Figure S1.**	**^{1}H NMR spectrum (600 MHz, MeOH-d_4) of hectoramide B (1).**	**S5**
**Figure S2.**	**^{1}H NMR spectrum of hectoramide B (1) with removal of solvent peaks using the Global Spectral Deconvolution (GSD) solvent suppression tool from MestReNova.**	**S6**
**Figure S3.**	**^{1}H-^{13}C HSQC spectrum (600 MHz, MeOH-d_4) of hectoramide B (1).**	**S7**
**Figure S4.**	**^{1}H-^{1}H COSY spectrum (600 MHz, MeOH-d_4) of hectoramide B (1).**	**S8**
**Figure S5.**	**^{1}H-^{13}C HMBC spectrum (600 MHz, MeOH-d_4) of hectoramide B (1).**	**S9**
**Figure S6.**	**Structures related to hectoramide B based on the SMART-NMR analysis of**	

the HSQC spectrum of hectoramide B (1). S10

Biosynthetic Gene Cluster Analysis of Hectoramide B (1)

Figure S7. Retrobiosynthetic scheme of hectoramide B (1) with the predicted modular organization of the hectoramide B biosynthetic gene cluster. S11
Figure S8. Deduced functions of proteins in *hca* biosynthetic gene cluster. S12
Figure S9. Phylogenetic tree of oxygen- and nitrogen-methyltransferase (MT) domains from cyanobacteria. S13
Figure S10. Methyltransferase domains from cyanobacteria used in the phylogenetic tree shown in Figure S10. S14
Figure S11. Adenylation domains used for sequence and structural alignment with HcaB-A domain. S15
Figure S12. Sequence alignment of hcaD and vatR terminating module. S16

LC-MS Analysis of Co- and Mono-cultures

Figure S13. Summary table of crude extracts obtained from co- and mono-cultures. S17
Figure S14. Analysis of Relative Abundance of Secondary Metabolites in Co- and Mono-culture. S18

Additional Analytical Data for Hectoramide B (1)

Figure S15. HR-MS spectra of hectoramide B (1). S19
Figure S16. UV spectrum of hectoramide B (1). S20
Figure S17. IR spectrum of hectoramide B (1). S21
Figure S18. LRESIMS of hectoramide B (1). S22

Candida auris **and** ***C. albicans*** **Susceptibility Testing of Hectoramide B (1)**

Figure S19. Protocol and tabulated results for susceptibility testing of hectoramide B (1) to *Candida auris* and *C. albicans*. S23

Table S1. NMR Data Summary for Hectoramide B (1).

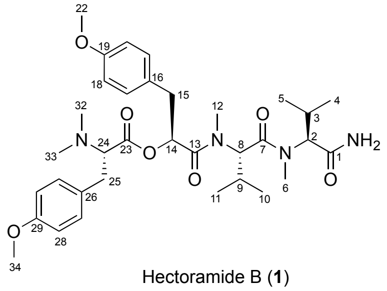

Hectoramide B (**1**)

Residue	Position	δC,[a] type	δH, type (J in Hz)	HMBC	COSY
N–Me Val(1)	1	173.6, C	–	–	–
	2	62.5, CH	4.62, d (11.1)	1, 3, 5, 6, 7	3
	3	26.8, CH	2.15, m	2, 4, 5	2, 4, 5
	4	19.1, CH_3	0.95, d (6.5)	2, 3, 5	3
	5	18.4, CH_3	0.69, d (6.6)	2, 3, 4	3
	6	30.8, CH_3	2.93, s	2, 7	–
N–Me Val(2)	7	171.8, C	–	–	–
	8	59.1, CH	5.08, d (10.7)	7, 9, 10, 12	9
	9	27.9, CH	2.27, m	8, 10, 11	8, 10, 11
	10	18.9, CH_3	0.89, d (6.3)	8, 9, 11	9
	11	18.0, CH_3	0.77, d (6.9)	8, 9, 10	9
	12	30.1, CH_3	2.92, s	8, 13	–
Mpla(3)	13	170.5, C	–	–	–
	14	72.8, CH	5.37, dd (8.8, 5.4)	13, 15, 16, 23	15a, 15b
	15a	36.5, CH_2	2.99, m	13, 14, 16, 17/21	14
	15b	–	3.03, m	13, 14, 16, 17/21	14
	16	128.4, C	–	–	–
	17/21	131.0, CH	7.22, d (8.4)	15, 16, 18/20, 19, 21/17	18/20
	18/20	114.2, CH	6.89, d (8.4)	16, 19, 20/18	17
	19	159.9, C	–	–	–
	22	55.0, CH_3	3.76, s	19	–
N,N,O Me3 Tyr(4)	23	170.3, C	–	–	–
	24	70.3, CH	3.45, dd (10.6, 5.4)	23, 25, 26, 32/33	25a,25b
	25a	35.2, CH_2	2.85, m	23, 24, 26, 27/31	24
	25b	–	2.89, m	23, 24, 26, 27/31	24
	26	129.2, C	–	–	–
	27/31	130.4, CH	6.81, d (8.4)	25, 26, 28/30, 29, 31/27	28/30
	28/30	114.2, CH	6.71, d (8.4)	26, 27/31, 29, 30/28	27/31

	29	159.3	–	–	–
	34	55.0, CH_3	3.73, s	29	–
	32/33	41.5 CH_3	2.40,[b] s	24, 33/32	–

[a] ^{13}C NMR chemical shifts were determined by correlations observed in the HSQC and HMBC spectra.
[b]A sharp singlet peak of lower intensity is present at 2.38 ppm, and likely represents a minor conformational isomer of hectoramide B.

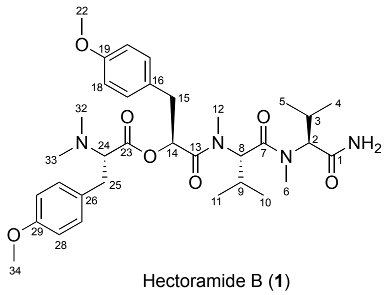

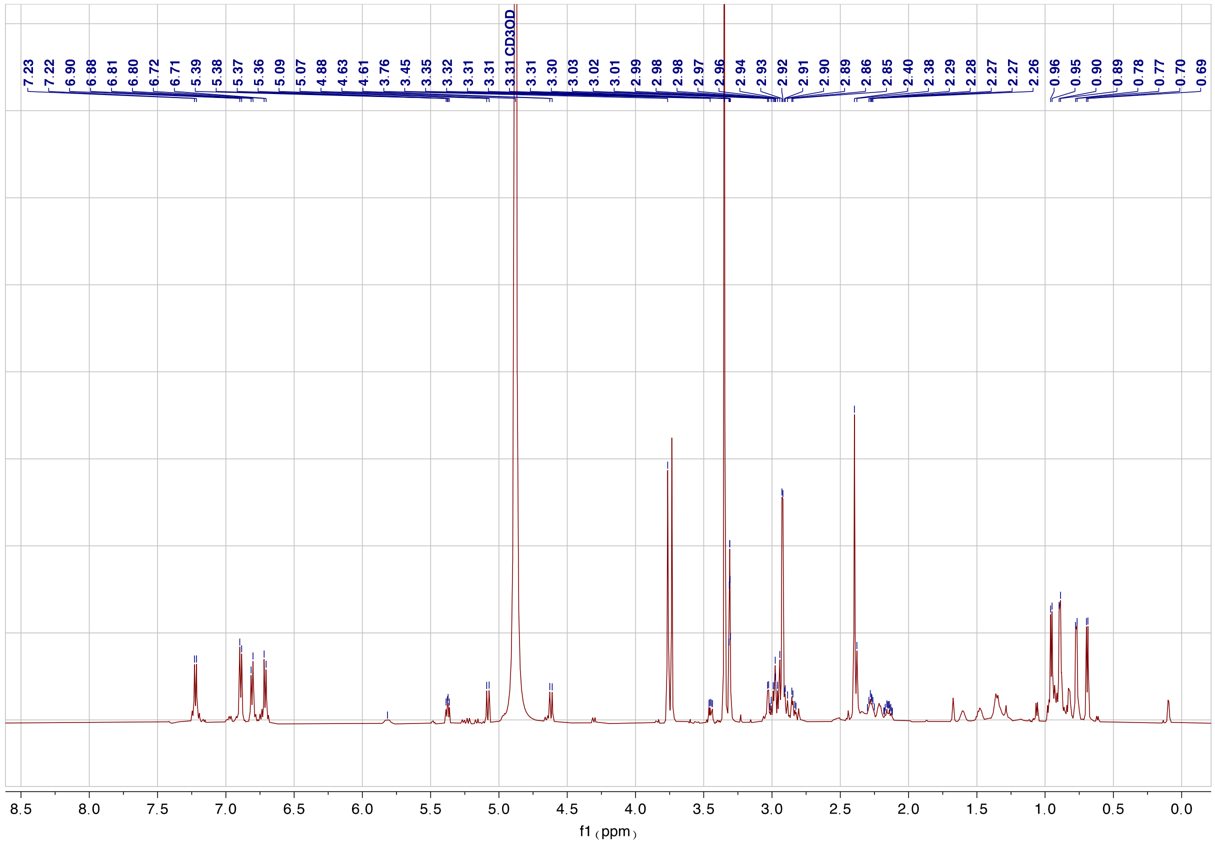

Figure S1. ^{1}H-NMR spectrum (600 MHz, MeOH-d_4) of hectoramide B (**1**).

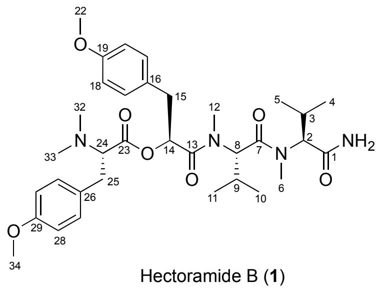

Hectoramide B (**1**)

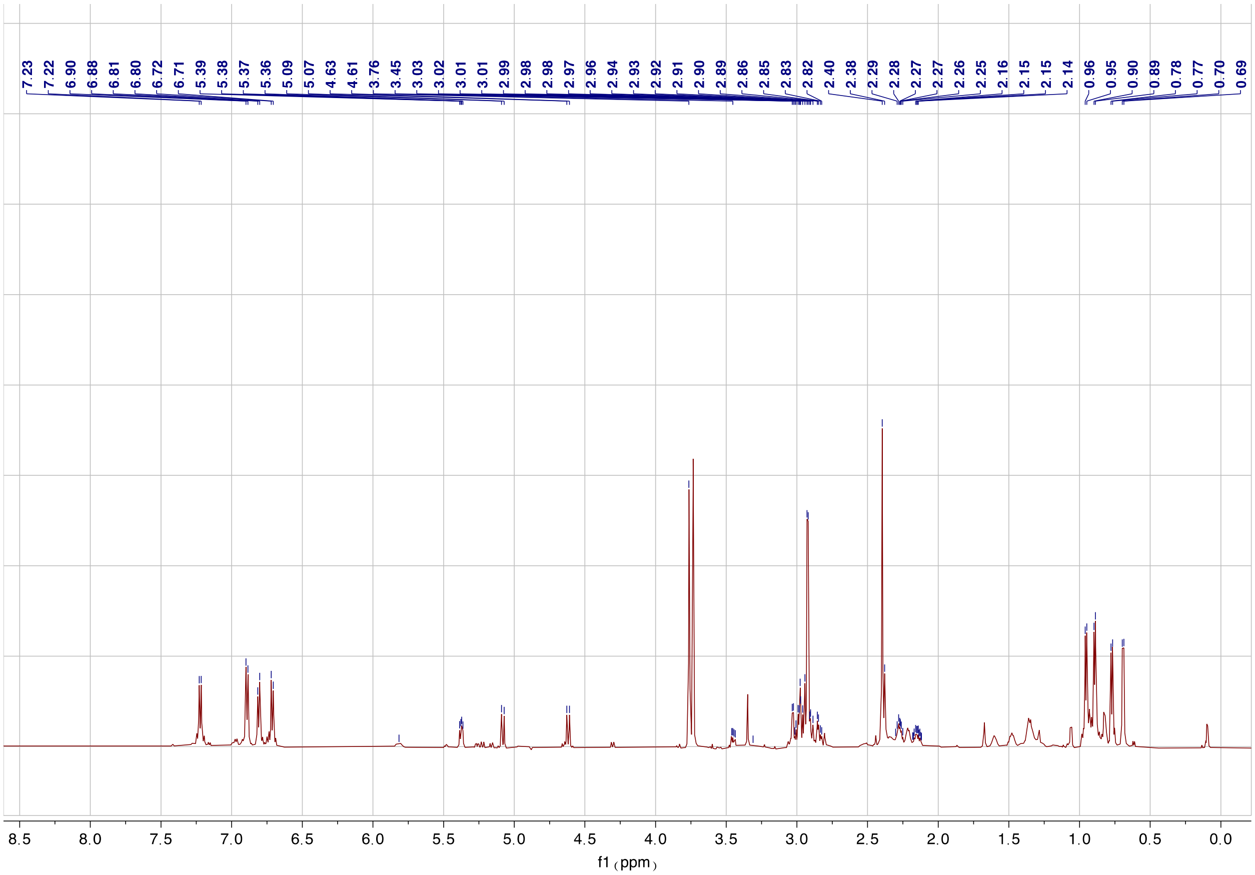

Figure S2. ^{1}H-NMR spectrum (600 MHz, MeOH-d_4) of hectoramide B (**1**) with removal of solvent peaks using the Global Spectral Deconvolution (GSD) solvent suppression tool from MestReNova.

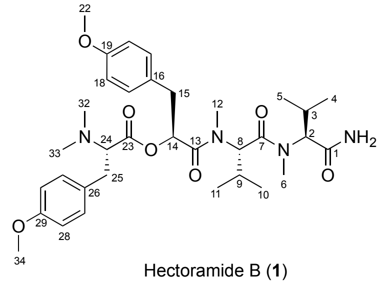

Hectoramide B (**1**)

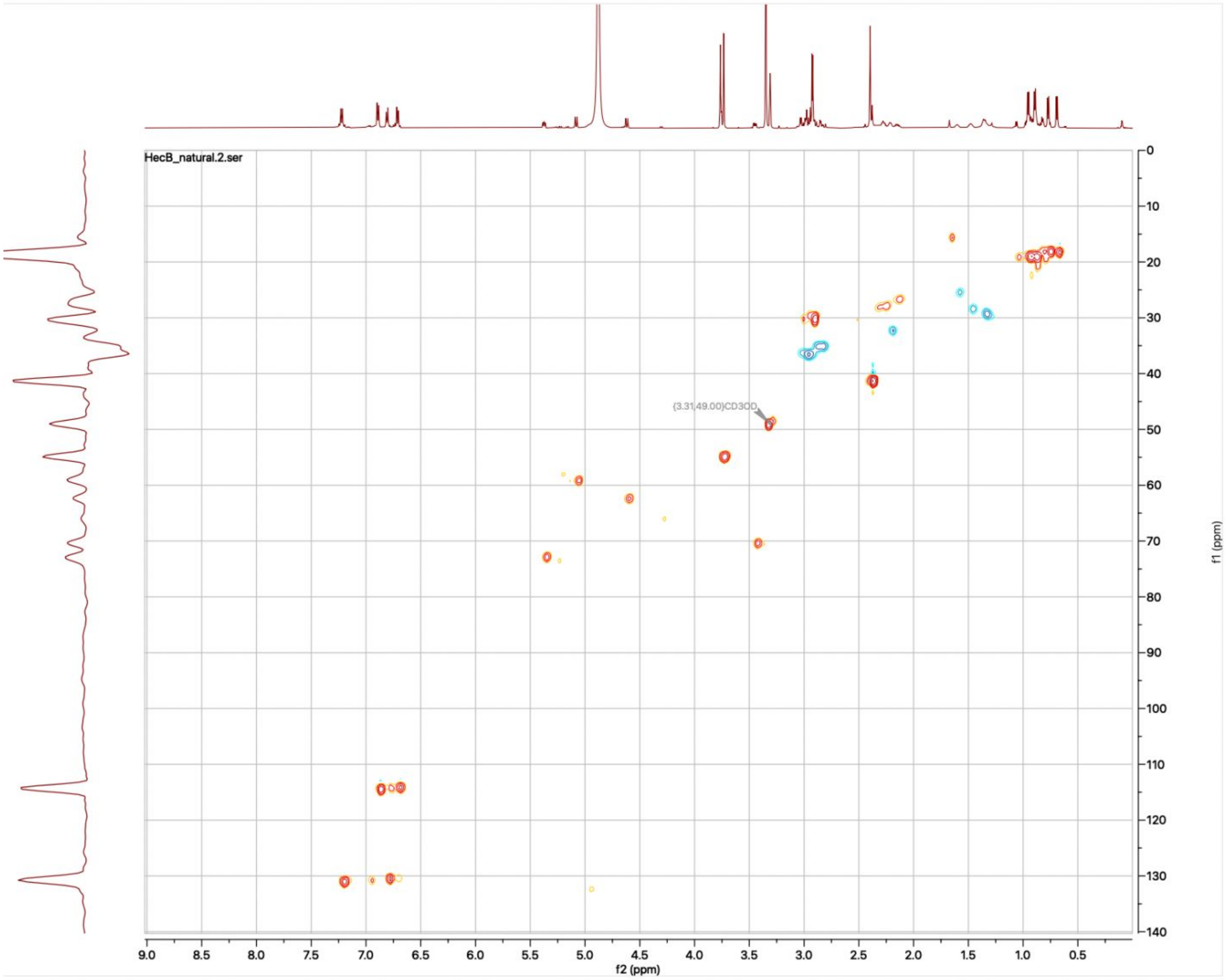

Figure S3. ^{1}H-^{13}C HSQC spectrum (600 MHz, MeOH-d_4) of hectoramide B (1).

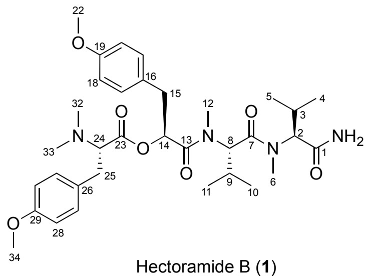

Hectoramide B (**1**)

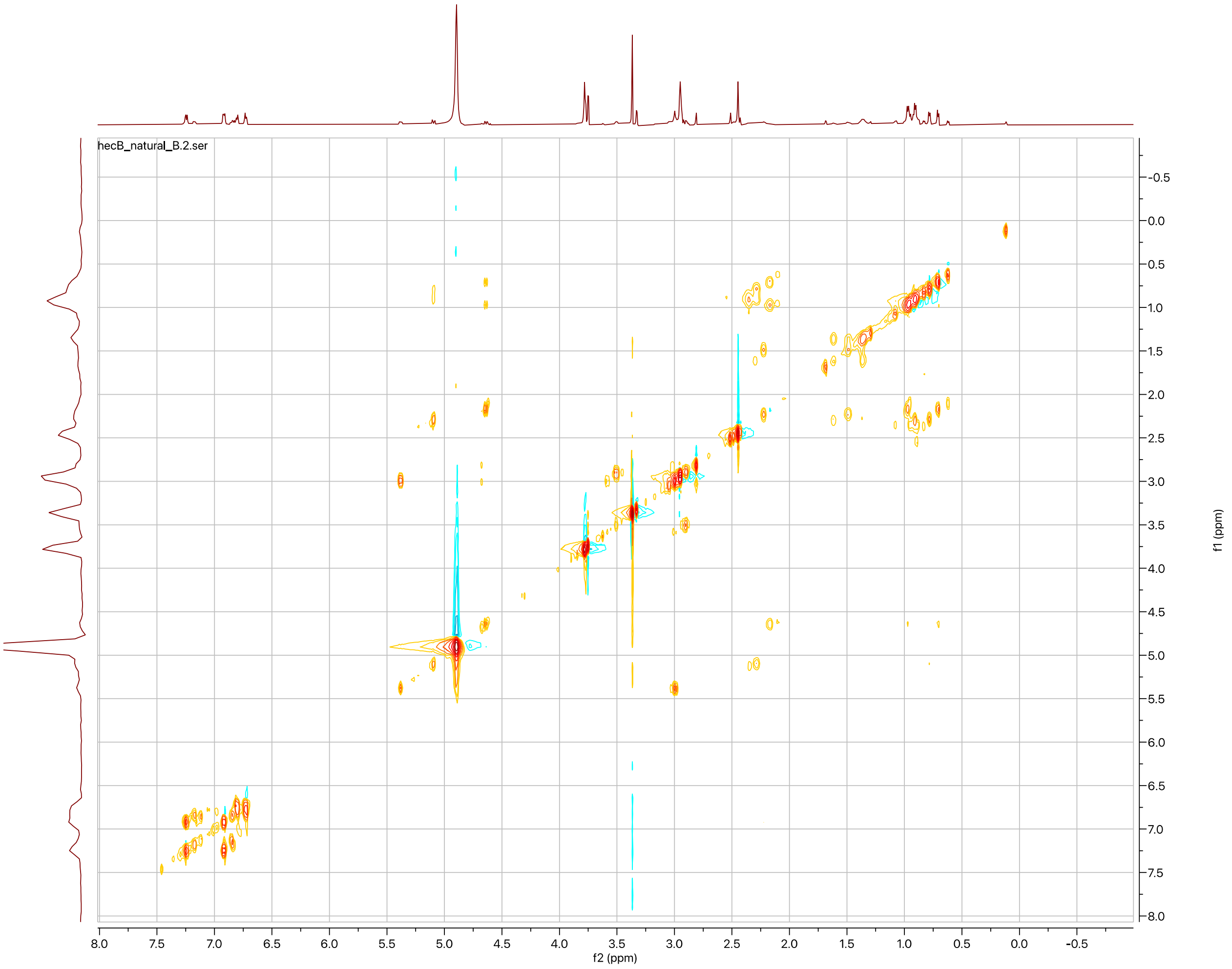

Figure S4. ^{1}H-^{1}H COSY spectrum (600 MHz, MeOH-d_4) of hectoramide B (1).

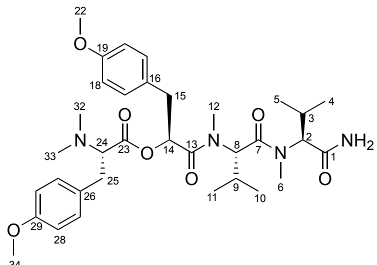

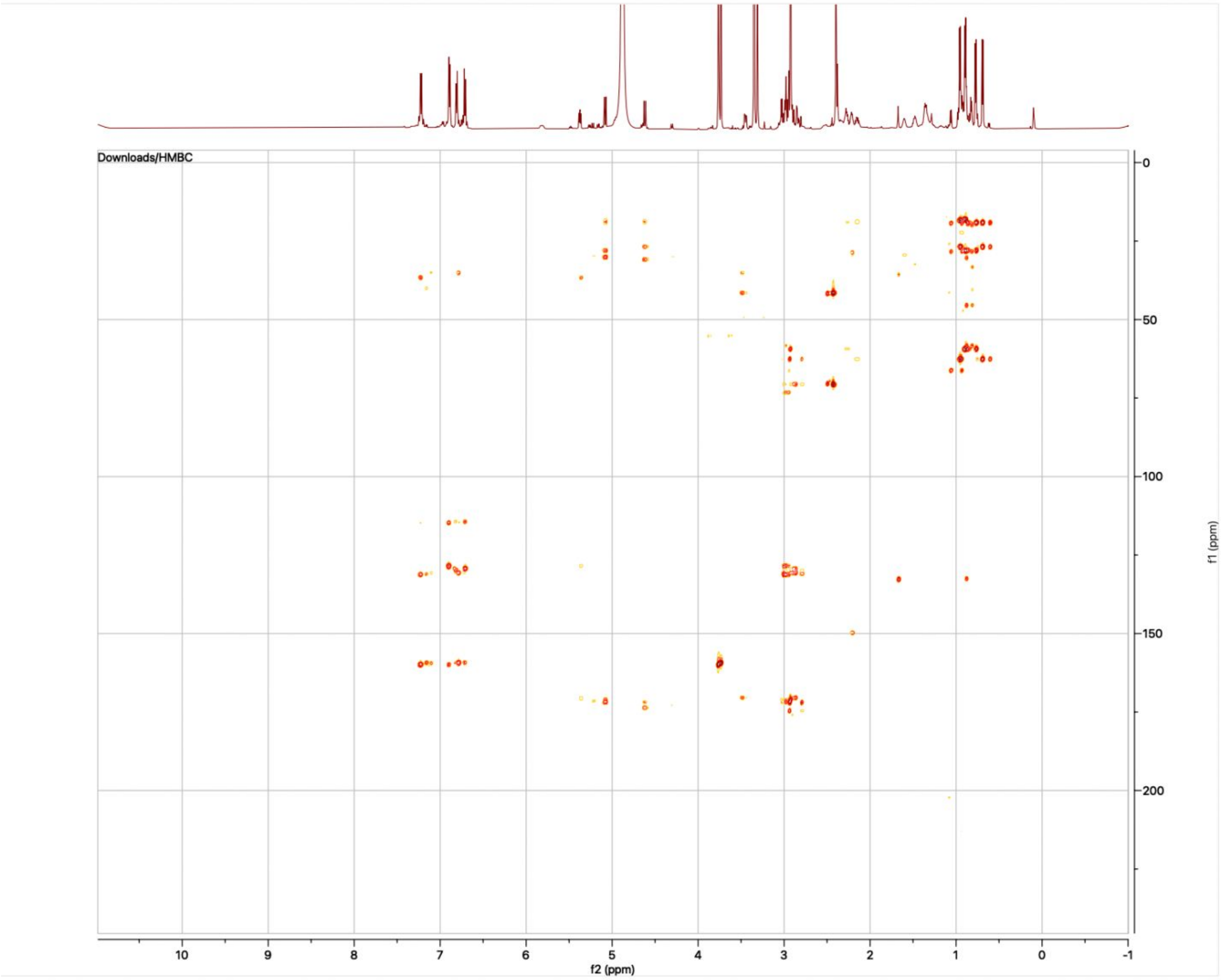

Figure S5. ^{1}H-^{13}C HMBC spectrum (600 MHz, MeOH-d_4) of hectoramide B (1).

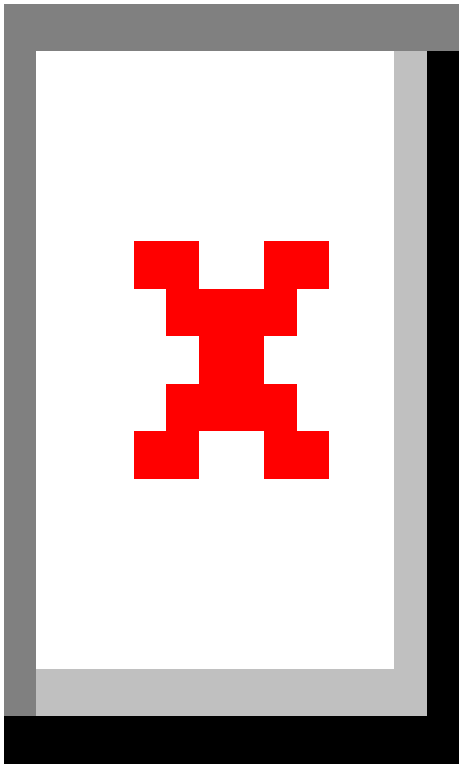

Figure S6. Structures related to hectoramide B based on the SMART-NMR analysis of the HSQC spectrum of hectoramide B (1).

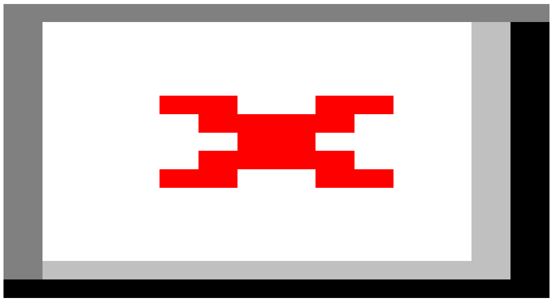

Figure S7. Retrobiosynthetic scheme of hectoramide B (1) with the predicted modular organization of the hectoramide B biosynthetic gene cluster. Circles represent enzymatic domains. C: condensation domain, TYR: adenylation domain for tyrosine, POPA: adenylation domain for 3-(4-hydroxyphenyl)-2-oxopropanoic acid, VAL: adenylation domain for valine, KR: ketoreductase domain, NMT: Nitrogen-methyltransferase domain, OMT: oxygen-methyltransferase domain, PCP: peptidyl-carrier protein, AMT: amidotransferase

Protein	Size (aa)	Catalytic domains	Proposed function	Sequence similarity	Identity/Similarity	Accession number	Query Cover
ORF 1	275	PGAP-1 like protein	hydrolase; acting on ester bonds	*Moorena bouilllonii*	89%, 95%	WP_229415337	99.6%
ORF 2	729	SARP family transcriptional regulator	Regulatory	*Moorena* sp. SIO3H5	73%, 80%	NEO70555	99.0%
ORF 3	73	Beta-keto acyl synthase	Beta-keto-acyl synthase	*Moorena producens*	100%, 100%	WP_083373734	98.6%
hcaA (Module 1)	1892	C, A(Tyr), OMT, NMT	NRPS; *N-N-*dimethyl-*O*-methyl tyrosine	LynB3, *Moorena bouillonii*	84%, 92%	BBU25144	98.4%
hcaB (Module 2)	2316	C, A(MPOPA), CAL, KR	NRPS; 2-hydroxy-3-(4-methoxyphenyl) propanoic acid	*Moorena producens*	100%, 100%	WP_071104444	100.0%
hcaC (Module 3)	1564	C, A(Val), NMT	NRPS; *N*-methyl-valine	LynB4, *Moorena bouillonii*	85%, 92%	BBU25144	98.0%
hcaD (Module 4)	1959	C, A(Val), NMT	NRPS; *N*-methyl-valine	LynB4, *Moorena bouillonii*	93%, 97%	WP_070391035	99.9%
ORF 4	130	hypothetical protein	unknown	*Myxacorys almedinensis*	67%, 81%	WP_162422484	99.3%
ORF 5	347	glycine betaine/L-proline ABC transporter substrate-binding protein	transmembrane transport	*Moorena sp. SIO1G6*	98%, 99%	NET62866	99.7%
ORF 6	372	glutamate 5-kinase	kinase	*Moorena sp. SIO1G6*	99%, 99%	NET62867	99.7%
ORF 7	303	MBL fold-metallo-hydrolase	hydrolase	*Moorena* sp. SIOASIH	99%, 100%	NEO41466	99.7%
ORF 8	60	hypothetical protein	unknown	*Moorena* sp. SIO3G5	77%, 83%	NEO95990	98.4%
ORF 9	276	hypothetical protein	unknown	*Moorena* sp. SIO3E8	95%, 98%	NEO15771	99.6%
ORF 10	56	hypothetical protein	unknown	*Moorena* sp. SIOASIH	98%, 100%	NEO41462	98.3%
ORF 11	446	Sugar binding lipoprotein	ABC transporter substrate-binding protein	*Moorena* sp. SIOASIH	96%, 97%	NEO41460	98.2%
ORF 12	294	sugar ABC transporter permease	transmembrane transport	*Moorena* sp. SIO2B7	99%, 100%	NES81698	99.7%
ORF 13	123	carbohydrate ABC transporter permease	transmembrane transport	*Moorena bouilllonii*	98%, 100%	WP_075896483	99.7%
ORF 14	123	hypothetical protein	unknown	*Moorena producens* 3L	99%, 100%	EGJ31142	99.2%
ORF 15	117	23S rRNA-intervening sequence protein	unknown	*Moorena* sp. SIO3A5	99%, 100%	NEP67679	99.2%
ORF 16	395	ABC transporter ATP-binding protein	transport	*Moorena* sp. SIO4A3	97%, 98%	NEO45280	99.8%

Figure S8. Deduced functions of the proteins in the *hca* biosynthetic gene cluster.

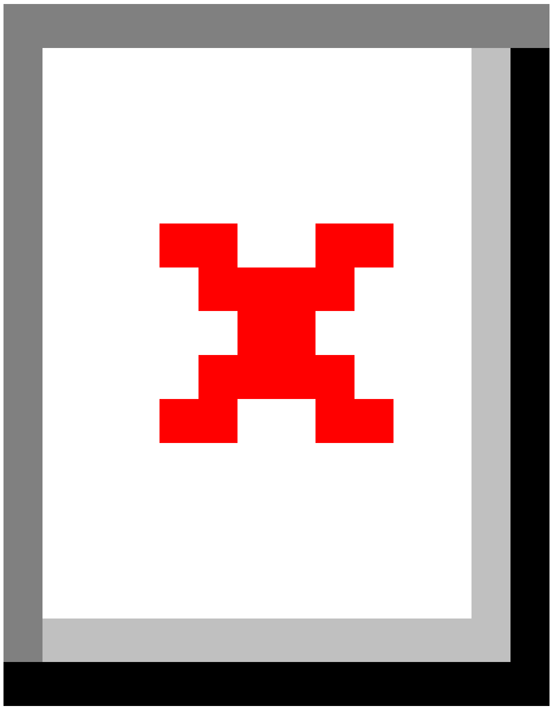

Figure S9. Phylogenetic tree of oxygen- and nitrogen-methyltransferase (MT) domains from cyanobacteria. This analysis identified the specificity of the two MT domains encoded by *hcaA*. OMT domain clades are outlined in blue. NMT domain clades are outlined in red.

Organism	Specificity	Genbank accession ID	MIBIG Accession ID	Natural product	Gene
Anabaena minutissima UTEX B 1613	NMT	MH325199.1	BGC0001952	minutissamide A	*puwA*
Anabaena sp. 90	NMT	GU174493.1	BGC0000302	Anabaenopeptin	*aptC*
Anabaena sp. 90	NMT	AY212249.1	BGC0001016	microcystin	*mycA*
Anabaena sp. Syke748	NMT	KJ502174.1	BGC0000369	hassalidin C	*hasY*
Anabaena sp. UHCC-0399	NMT	MH325200.1	BGC0001953	puwainaphycin F	*puwA*
Brasilonema sp. CT11	NMT	MT670293.1	BGC0002512	Anabaenopeptin 788	*aptC*
Cylindrospermum alatosporum CCALA 988	NMT	KM078884.1	BGC0001125	puwainaphycin A	*puwA*
Cylindrospermum moravicum CCALA 993	NMT	MH325197.1	BGC0001950	puwainaphycin F	*puwA*
Fischerella sp. PCC 9431	NMT	NZ_KE650771.1	BGC0001467	hapalosin	*FIS9431_RS32925*
Fischerella sp. PCC 9431	NMT	NZ_KE650771.1	BGC0002624	fischerazole A	*FIS9431_RS33740*
Fischerella thermalis	OMT	WP_102182194.1	-	-	-
Fischerella thermalis	OMT	WP_102176734.1	-	-	-
Fischerella thermalis	OMT	WP_102172701.1	-	-	-
Fischerella thermalis	OMT	WP_009460361.1	-	-	-
Fischerella thermalis CCMEE 5319	OMT	PMB16080.1	-	-	-
Lyngbya majuscula	NMT	AY588942.1	BGC0000384	lyngbyatoxin	*LtxA*
Lyngbya majuscula	NMT	AF516145.1	BGC0000962	barbamide	*barG*
Microcystis aeruginosa	OMT	WP_002738215.1	-	-	-
Microcystis aeruginosa	OMT	BCU10658.1	-	-	-
Microcystis aeruginosa 4A3	OMT	AZP89422.1	-	-	-
Microcystis aeruginosa FACHB-524	OMT	ROI01679.1	-	-	-
Microcystis aeruginosa K-139	NMT	AB481215.1	BGC0001018	micropeptin K139	*mcnC*
Microcystis aeruginosa Ma_MB_F-20061100_S20	OMT	TRU39906.1	-	-	-
Microcystis aeruginosa Ma_MB_F-20061100_S20*D*	OMT	TRU37805.1	-	-	-
Microcystis aeruginosa NIES-2549	OMT	AKE63547.1	-	-	-
Microcystis aeruginosa NIES-3787	OMT	GCL44916.1	-	-	-
Microcystis aeruginosa NIES-3806	OMT	GCL53376.1	-	-	-
Microcystis aeruginosa NIES-3806	OMT	GCL49408.1	-	-	-
Microcystis aeruginosa NIES-3807	OMT	GCL56935.1	-	-	-
Microcystis aeruginosa NIES-98	OMT	ODV39796.1	-	-	-
Microcystis aeruginosa PCC 7806	NMT	AF183408.1	BGC0001017	microcystin LR	*mycA*
Microcystis sp. NIVA-CYA 172/5	NMT	DQ075244.1	BGC0000332	Cyanopeptolin	*mcnC*
Moorea producens JHB	NMT	KY315923.1	BGC0001560	Cryptomaldamide	*cpmB*
Moorena bouillonii	NMT	GA0081470	-	-	-
Moorena producens	NMT	OLT68856.1	-	-	-
Moorena producens 3L	OMT	EGJ32162.1	-	-	-
Moorena producens 3L	OMT	EGJ32090.1	-	-	-
Moorena producens ASI16Jul14-2	NMT	MK618714.1	BGC0002296	Vatiamide A,B	*VatN - MT1*
Moorena producens ASI16Jul14-2	NMT	MK618714.1	BGC0002296	Vatiamide A,B	*VatR*
Moorena producens ASI16Jul14-2	NMT	MK618714.1	BGC0002296	Vatiamide A,B	*VatS*
Moorena producens ASI16Jul14-2	OMT	MK618714.1	BGC0002296	Vatiamide A,B	*VatN - MT2*
Nostoc linckia	OMT	WP_242052790.1	-	-	-
Nostoc linckia	OMT	WP_190659225.1	-	-	-
Nostoc linckia	OMT	WP_099068175.1	-	-	-
Nostoc linckia	OMT	WP_096540073.1	-	-	-
Nostoc linckia	OMT	WP_096538766.1	-	-	-
Nostoc punctiforme	OMT	WP_190950119.1	-	-	-
Nostoc punctiforme	OMT	WP_190948102.1	-	-	-
Nostoc punctiforme PCC 73102	NMT	CP001037.1	BGC0001479	enopeptin NZ857, nostai	*Npun_F2462*
Nostoc punctiforme PCC 73102	OMT	ACC83904.1	-	-	-
Nostoc punctiforme PCC 73102	OMT	ACC82073.1	-	-	-
Nostoc sp. 73.1	NMT	JF342711.1	BGC0000396	nodularin	*ndaA*
Nostoc sp. ATCC 53789	OMT	EF159954.1	BGC0000975	cryptophycin-327	*crpC*
Nostoc sp. CENA543	NMT	MF668122.1	BGC0001705	nodularin	*ndaA*
Nostoc sp. UHCC 0702	NMT	CP071065.1	BGC0002572	heinamide A1	*JYQ62_16355*
Okeania hirsuta	NMT	MK142793.1	BGC0001971	Malyngamide I	*MgcJ*
Oscillatoriales	OMT	TAG39103.1	-	-	-
Oscillatoriales	OMT	TAF21875.1	-	-	-
Oscillatoriales	OMT	TAE13988.1	-	-	-
Oscillatoriales cyanobacterium	OMT	TAE67097.1	-	-	-
Phormidium sp. LP904c	NMT	MK870090.1	BGC0002297	MC-LR	*mycA*
Planktothrix agardhii	OMT	CAD29800.2	-	-	-
Planktothrix agardhii NIVA-CYA 126/8	NMT	AJ441056.1	BGC0001015	microcystin	*mycA*
Planktothrix aghardhii	NMT	EF672686.1	BGC0000301	Anabaenopeptin 908	*apnC*
Planktothrix aghardhii NIVA-CYA 116	NMT	DQ837301.1	BGC0000331	Cyanopeptolin	*ociB*
Planktothrix serta PCC 8927	NMT	LT546031.1	BGC0001614	hassalidin E	*hasY*
Streptomyces anulatus	NMT	HM038106.1	BGC0000296	actinomycin D	*acmC*
Tistrella mobilis KA081020-065	OMT	CP003239.1	BGC0000985	didemnin	*didJ*

Figure S10. Methyltransferase domains from cyanobacteria used to build the phylogenetic tree shown in Figure S9. Sequences were prepared from NCBI database and MiBIG database.

Gene	Specificity	Organism	Genbank accession ID	MIBIG Accession ID	Natural product	PDB Accesion
GrsA	Phenylalanine	*Aneurinibacillus migulanus*	LGUG01000004.1	BGC0002122	gramicidin S	-
TycC	Tyrosine	*Brevibacillus brevis* NBRC 100599	AP008955.1	BGC0000452	tyrocidine	-
FenB	Tyrosine	*Bacillus velezensis* FZB42	CP000560.1	BGC0001095	fengycin	-
AusA	Tyrosine	*Staphylococcus aureus* subsp. aureus str. JKD6008	CP002120.1	BGC0000308	aureusimine A	-
AprJ	Tyrosine	*Moorena bouillonii* bAprat14	MG890637.1	BGC0002542	apratoxin A	-
StaC	Tyrosine	*Streptomyces toyocaensis*	U82965.2	BGC0000290	A-47934	-
TycA	Phenylalanine	*Brevibacillus brevis* NBRC 100599	AP008955.1	BGC0000452	tyrocidine	-
TycB	Phenylalanine	*Brevibacillus brevis* NBRC 100599	AP008955.1	BGC0000452	tyrocidine	-
CesB	α-keto-isovaleric acid	*Bacillus cereus*	DQ360825.1	BGC0000320	cereulide	-
CesA	α-keto-isovaleric acid	*Bacillus cereus*	DQ360825.1	BGC0000320	cereulide	-
LgrA (mutant)	α-keto-isocaproic acid	*Brevibacillus parabrevis*	-	-	Linear gramicidin	6ULZ

Figure S11. Adenylation domains used for sequence and structural alignment with HcaB adenylation domain.

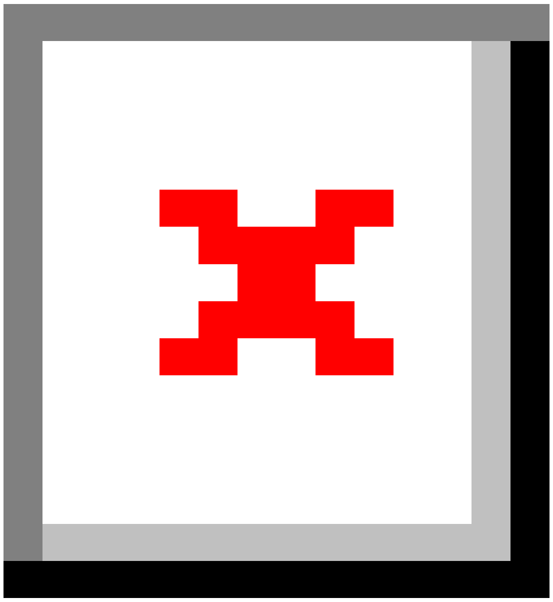

Figure S12. Sequence alignment of hcaD and vatR terminating module. (a) Both pathways are predicted to encode enzymes that incorporate an *N*-methyl valinamide terminus. While these two pathways show 72% identity when aligning all 1885 amino acids, they are 93.0% identical for the 415 residues of the proposed terminating ammonolysis domain. Identical residues are highlighted in red, similar residues are outlined in blue and presented in red text. (b) Sequence alignment in gene graphic format

SAMPLE	EXTRACT (g)	vMeOH 0.5 mg/mL	1 mg/mL	2 mg/mL	Concentration (mg/mL)
JHB 1	0.0301	15.05	7.525	-	1
JHB 2	0.0192	9.6	-	-	0.5
JHB 3	0.0191	9.55	-	-	0.5
CA 1	0.0464	23.2	11.6	5.8	2
CA 2	0.0739	36.95	18.475	9.2375	2
CA 3	0.0061	3.05	-	-	0.5
JHB CA 1	0.0142	7.1	-	-	0.5
JHB CA 2	0.0171	8.55	-	-	0.5
JHB CA 3	0.0249	12.45	6.225	-	1
JHB CA MEDIA 1	0.0111	5.55	-	-	0.5
JHB CA MEDIA 2	0.0015	0.75	-	-	0.5
JHB CA MEDIA 3	0.0324	16.2	8.1	-	1
SWBG11 CONTROL	0.0672	33.6	16.8	8.4	2

Figure S13. Summary table of crude extracts obtained from co- and mono-cultures. JHB: *Moorena producens* JHB; CA: *Candida albicans*

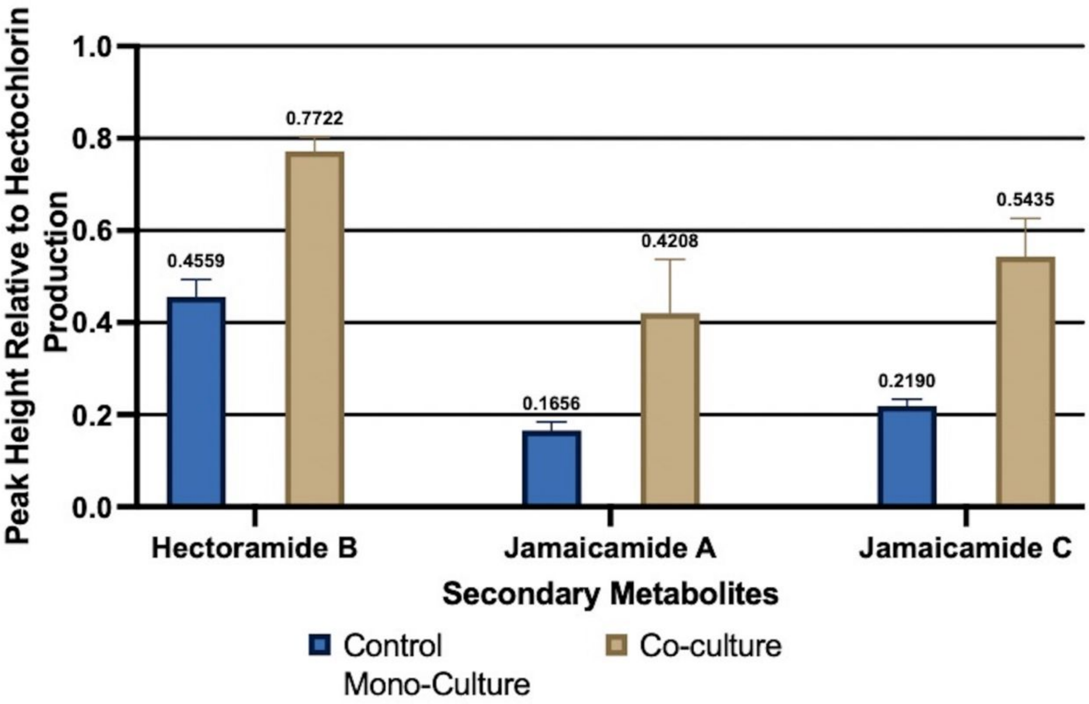

Figure S14. Analysis of Relative Abundance of Secondary Metabolites in Co- and Mono-cultures. Significant increase in the production of hectoramide B as well as two known, bioactive jamaicamides when normalized to the hectochlorin content in each sample. Hectoramide A (**2**) was not detected in these experiments.

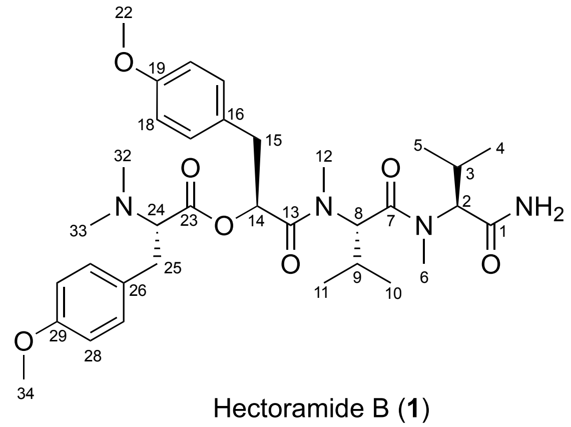

Hectoramide B (**1**)

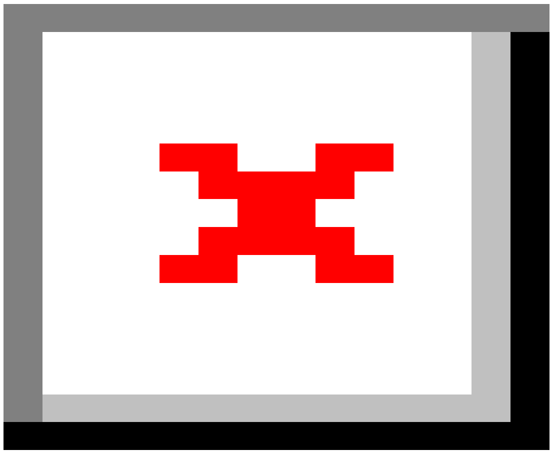

Figure S15. HRMS spectra of hectoramide B (1).

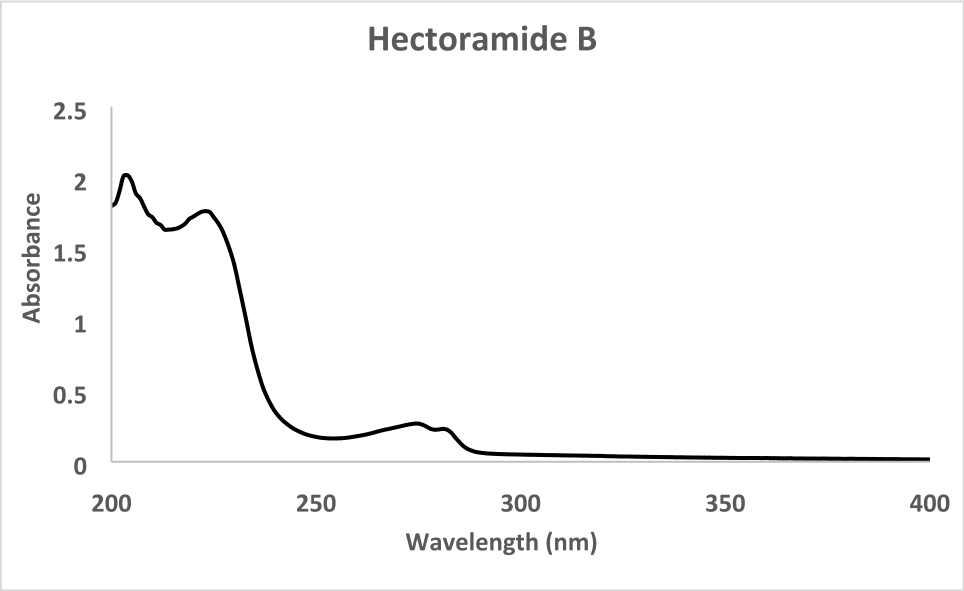

Figure S16. UV spectrum of hectoramide B (1).

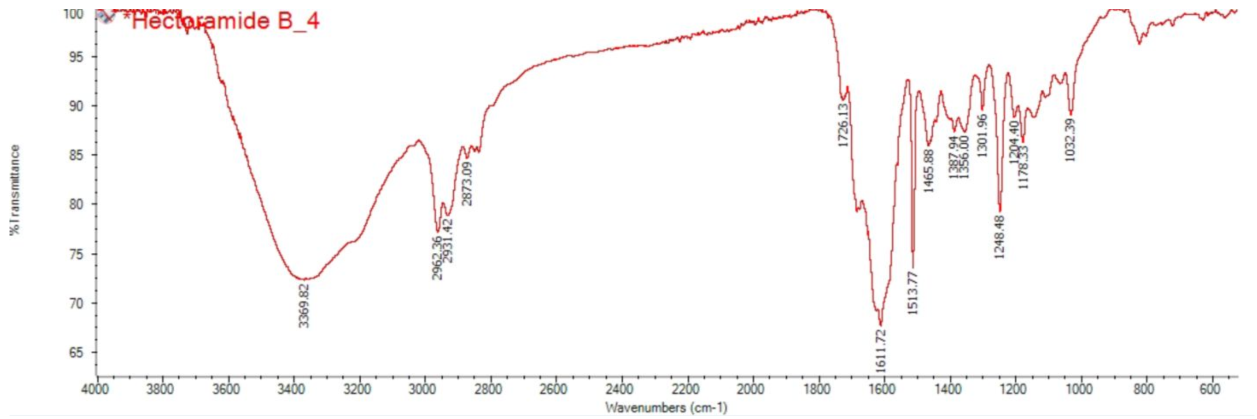

Figure S17. IR spectrum of hectoramide B (1).

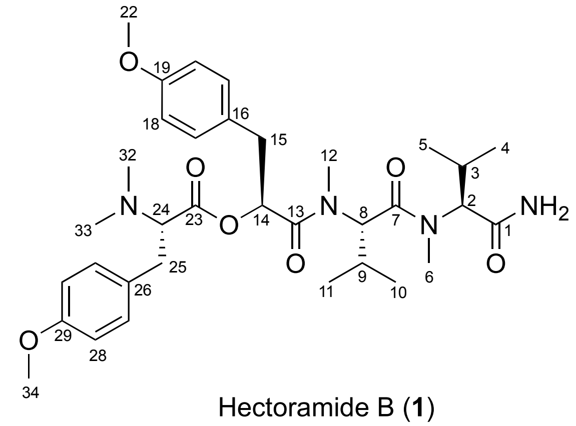

Hectoramide B (**1**)

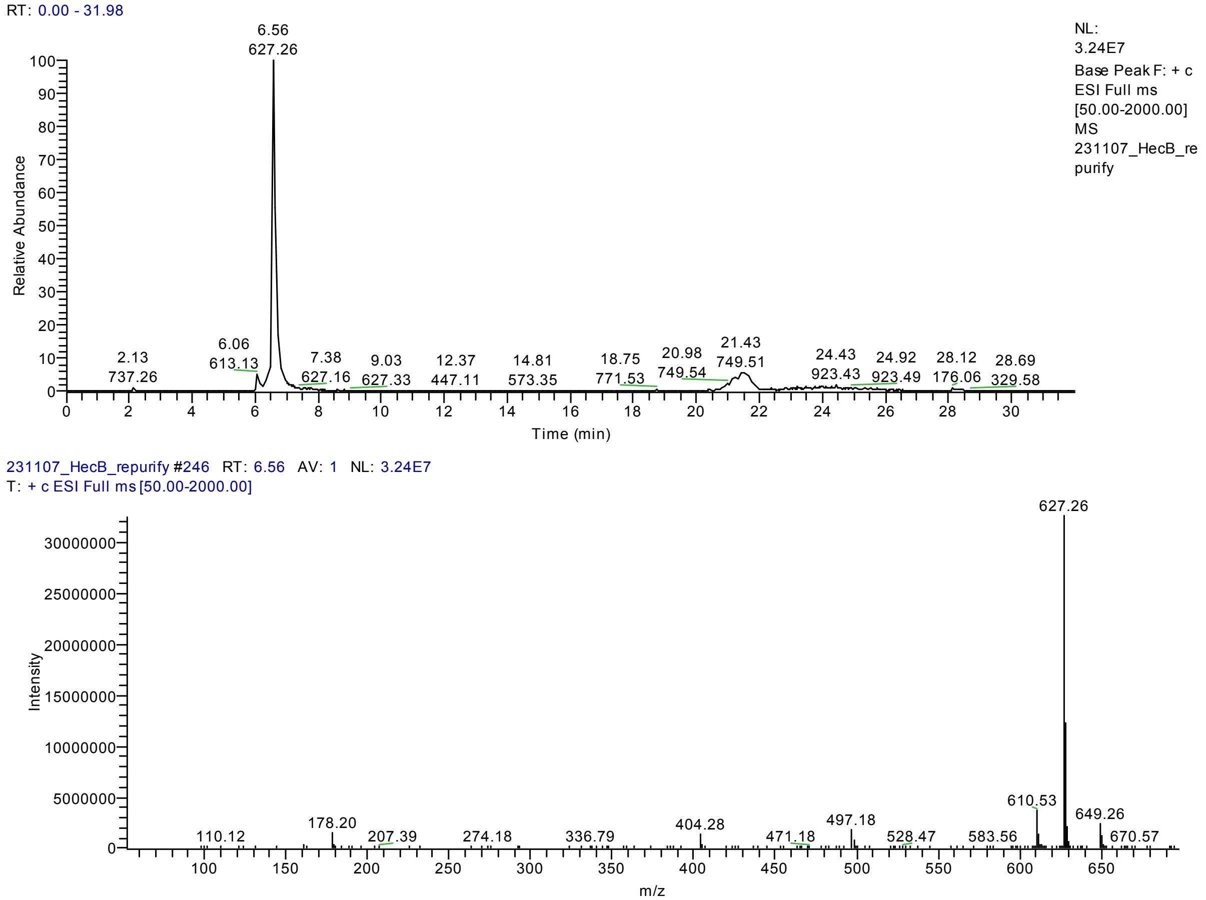

Figure S18. LRESIMS of hectoramide B (1).

Candida auris **and** ***C. albicans*** **Susceptibility Testing of Hectoramide A (1)**

Figure S19. Protocol and tabulated results for susceptibility testing of hectoramide B (1) to *Candida auris* and *C. albicans*

MICs to the two *Candida* species were determined following the guidelines of the European Committee on Antimicrobial Susceptibility Testing (EUCAST).[1] *C. auris* AR390 and *C. albicans* AR761 strains were grown in Yeast Peptone Dextrose overnight at 30 ^{o}C with shaking. The inoculum was prepared according to EUCAST methods, with modifications as follows: RPMI 1640 (US Biological R8998-07) was supplemented with 2% glucose and buffered with morpholinepropanesulfonic acid (MOPS) adjusted to pH 7.0. Hectoramide B (**1**) was serially diluted in a 96-well plate and added at a final starting concentration of 128 μg/ml. After 24 h the absorbance was read at 530 nm using an Enspire Alpha plate reader (Perkin Elmer).

The susceptibility tests indicate that hectoramide B has an MIC greater than 128 μg/mL to both *C. albicans* and *C. auris*.

C. albicans: AR761
C. auris: AR390

**DMSO + *C. albicans***												
									1.085	1.321	1.326	Yeast only
									0.082	0.083	0.082	Media only
0.294	1.181	1.205	1.168	1.226	1.168	1.208	1.221	1.139	1.173	1.111	1.231	
1.186	1.213	1.218	1.203	1.202	1.182	1.16	1.258	1.236	1.211	1.212	1.236	Yeast + DMSO
1.172	1.085	1.203	1.255	1.208	1.233	1.203	1.215	1.028	1.231	1.186	1.25	
***C. albicans* Rep #1**												
									1.272	1.291	1.253	Yeast only
									0.084	0.086	0.083	Media only
**128**	**64**	**32**	**16**	**8**	**4**	**2**	**1**	**0.5**	**0.25**	**0.125**	**0.0625**	Compound conc (μg/mL)
1.357	1.336	1.304	1.297	1.268	1.259	1.244	0.674	1.13	1.184	1.112	1.196	
1.367	1.334	1.314	1.307	1.271	1.273	1.214	0.874	1.18	1.181	1.186	1.185	Yeast + compound
1.309	1.262	0.805	0.573	0.611	0.495	0.644	0.609	1.305	1.211	1.246	1.233	
***C. albicans* Rep #2**												
									1.139	1.162	1.124	Yeast only

									0.086	0.084	0.085	Media only
**128**	**64**	**32**	**16**	**8**	**4**	**2**	**1**	**0.5**	**0.25**	**0.125**	**0.0625**	Compound conc (μg/mL)
1.203	1.255	1.224	1.178	1.16	1.108	1.054	0.685	1.078	1.091	1.016	1.066	
1.253	1.234	1.2	1.173	1.081	1.092	1.087	0.438	1.07	1.06	1.055	1.068	Yeast + compound
1.226	1.228	0.562	0.632	0.62	0.744	1.065	0.537	0.771	1.099	1.102	1.069	
C. albicans **Rep #3**												
									1.051	1.035	1.069	Yeast only
									0.084	0.084	0.087	Media only
**128**	**64**	**32**	**16**	**8**	**4**	**2**	**1**	**0.5**	**0.25**	**0.125**	**0.0625**	Compound conc (μg/mL)
1.315	1.27	1.259	1.22	1.212	1.146	1.186	1.174	1.137	1.141	1.076	1.171	
1.29	1.258	1.245	1.232	1.235	1.179	1.146	1.14	1.135	1.138	1.142	1.144	Yeast + compound
1.293	1.264	1.249	1.213	1.213	1.189	1.171	1.138	1.103	1.117	1.087	1.124	

C. auris

**DMSO +** ***C. auris***												
									0.608	0.615	0.609	Yeast only
									0.085	0.085	0.085	Media only
												DMSO
0.694	0.679	0.653	0.65	0.644	0.644	0.629	0.621	0.646	0.628	0.62	0.523	
0.67	0.66	0.6	0.638	0.623	0.629	0.621	0.617	0.619	0.624	0.623	0.508	Yeast + DMSO
0.647	0.675	0.598	0.651	0.639	0.63	0.631	0.616	0.617	0.631	0.635	0.523	
C. auris **Rep #1**												
									1.491	1.559	1.598	Yeast only
									0.087	0.085	0.086	Media only
**128**	**64**	**32**	**16**	**8**	**4**	**2**	**1**	**0.5**	**0.25**	**0.125**	**0.0625**	Compound conc (μg/mL)
0.697	0.823	0.709	0.663	0.636	0.6	0.538	0.571	0.607	0.543	0.595	0.538	
0.821	0.859	0.743	0.675	0.588	0.611	0.601	0.571	0.518	0.579	0.58	0.601	Yeast + compound
0.843	0.902	0.768	0.647	0.629	0.625	0.586	0.597	0.611	0.537	0.58	0.584	

***C. auris* Rep #2**												
									1.541	1.586	1.601	Yeast only
									0.086	0.087	0.087	Media only
**128**	**64**	**32**	**16**	**8**	**4**	**2**	**1**	**0.5**	**0.25**	**0.125**	**0.0625**	Compound conc (μg/mL)
0.654	0.78	0.778	0.659	0.636	0.621	0.588	0.524	0.569	0.57	0.599	0.601	
0.786	0.901	0.769	0.659	0.591	0.597	0.582	0.588	0.568	0.547	0.596	0.603	Yeast + compound
0.837	0.912	0.793	0.699	0.642	0.626	0.576	0.529	0.599	0.602	0.602	0.598	
***C. auris* Rep #3**												
									0.56	0.57	0.607	Yeast only
									0.084	0.085	0.087	Media only
**128**	**64**	**32**	**16**	**8**	**4**	**2**	**1**	**0.5**	**0.25**	**0.125**	**0.0625**	Compound conc (μg/mL)
0.916	0.933	0.841	0.734	0.677	0.613	0.625	0.588	0.638	0.617	0.666	0.589	
0.906	0.936	0.807	0.734	0.654	0.591	0.624	0.569	0.627	0.639	0.621	0.597	Yeast + compound
0.929	0.996	0.887	0.796	0.685	0.629	0.645	0.627	0.65	0.601	0.678	0.639	

NB: Since the Hectoramide B was resuspended in DMSO, DMSO was used as a vehicle control.

References

1. Guinea, J.; Meletiadis, J.; Arikan-Akdagli, S.; Giske, C.; Muehlethaler, K.; Arendrup, M.C. and the Subcommittee on Antifungal Susceptibility Testing (AFST) of the ESCMID European Committee for Antimicrobial Susceptibility Testing (EUCAST). **2023**. Method for the determination of broth dilution minimum inhibitory concentrations of antifungal agents for yeasts. EUCAST Definitive Document E.Def 7.4
